# DEG 15, an update of the Database of Essential Genes that includes built-in analysis tools

**DOI:** 10.1093/nar/gkaa917

**Published:** 2020-10-23

**Authors:** Hao Luo, Yan Lin, Tao Liu, Fei-Liao Lai, Chun-Ting Zhang, Feng Gao, Ren Zhang

**Affiliations:** Department of Physics, School of Science, Tianjin University, Tianjin 300072, China; Department of Physics, School of Science, Tianjin University, Tianjin 300072, China; Department of Physics, School of Science, Tianjin University, Tianjin 300072, China; Department of Physics, School of Science, Tianjin University, Tianjin 300072, China; Department of Physics, School of Science, Tianjin University, Tianjin 300072, China; Department of Physics, School of Science, Tianjin University, Tianjin 300072, China; Frontiers Science Center for Synthetic Biology and Key Laboratory of Systems Bioengineering (Ministry of Education), Tianjin University, Tianjin 300072, China; Center for Molecular Medicine and Genetics, School of Medicine, Wayne State University, Detroit, MI 48201, USA

## Abstract

Essential genes refer to genes that are required by an organism to survive under specific conditions. Studies of the minimal-gene-set for bacteria have elucidated fundamental cellular processes that sustain life. The past five years have seen a significant progress in identifying human essential genes, primarily due to the successful use of CRISPR/Cas9 in various types of human cells. DEG 15, a new release of the Database of Essential Genes (www.essentialgene.org), has provided major advancements, compared to DEG 10. Specifically, the number of eukaryotic essential genes has increased by more than fourfold, and that of prokaryotic ones has more than doubled. Of note, the human essential-gene number has increased by more than tenfold. Moreover, we have developed built-in analysis modules by which users can perform various analyses, such as essential-gene distributions between bacterial leading and lagging strands, sub-cellular localization distribution, enrichment analysis of gene ontology and KEGG pathways, and generation of Venn diagrams to compare and contrast gene sets between experiments. Additionally, the database offers customizable BLAST tools for performing species- and experiment-specific BLAST searches. Therefore, DEG comprehensively harbors updated human-curated essential-gene records among prokaryotes and eukaryotes with built-in tools to enhance essential-gene analysis.

## INTRODUCTION

**Figure 1.**
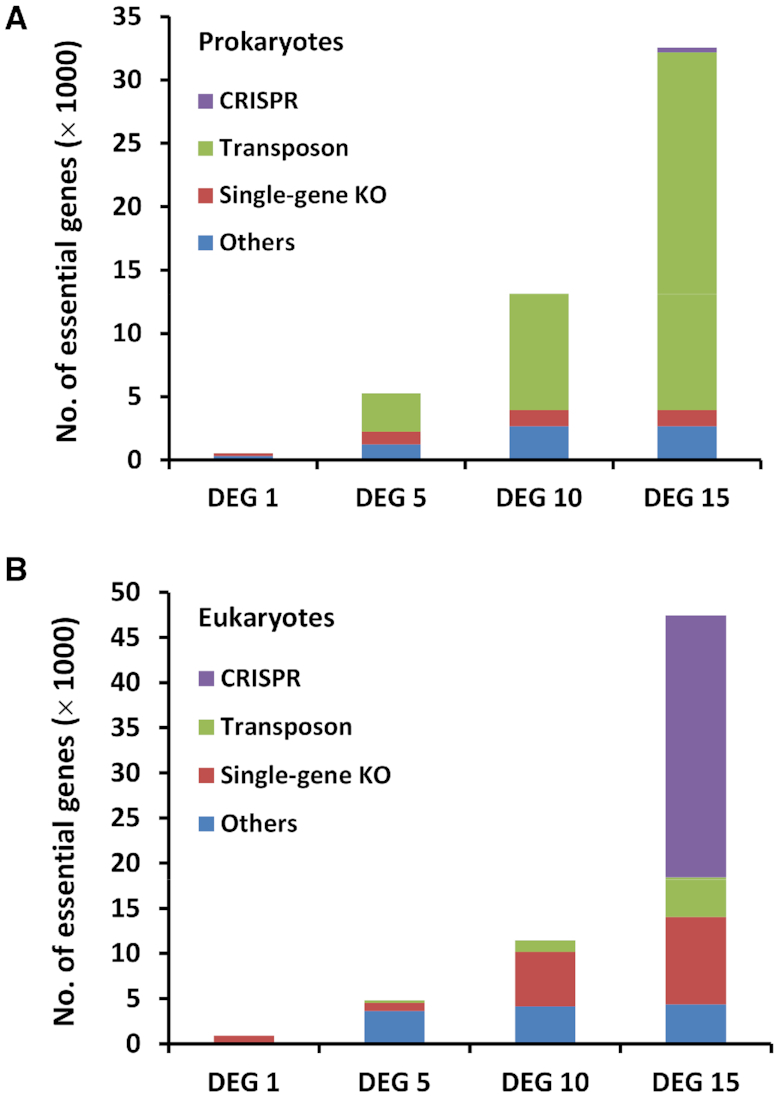
The development of DEG. The number of essential-gene records for (**A**) prokaryotes and (**B**) eukaryotes in DEG with different versions. The stacked bars show the number of records according to the experimental methods.

Essential genes refer to genes required for a cell or an organism to survive under certain conditions (1,2). The research on the determination of essential genes has attracted significant attention in the past decade, due to its theoretical implications and practical uses. Studies of genome-wide gene essentiality screenings have elucidated fundamental cellular processes that sustain life (2). We created DEG, a Database of Essential Genes in 2003 (3), a time when the genome-scale gene essentiality screening was still not available. The development of DEG parallels with the development of the essential-gene field. Significant progress has been made in performing genome-wide essentiality screenings among diverse species, primarily due to technological developments. We subsequently published DEG 5, which included essential genes of both bacteria and eukaryotes (4), and DEG 10, which included both protein-coding genes and non-coding genomic elements (5). Since 2014, when DEG 10 was published (5), significant progress has been made mainly owing to the invention of CRISPR/Cas9 (6,7) and the widespread use of Tn-seq (8,9). To accommodate the progress in essential-gene studies, we created DEG 15, which, compared to DEG 10, provides two major updates:

1. The number of essential-gene entries has significantly increased. Specifically, compared to DEG 10, the number of eukaryotic essential genes has increased by more than fourfold, and that of prokaryotic ones has more than doubled. Of note, the human essential-gene number has increased by more than ten-fold. Figure 1 shows the number of essential gene records in different versions of DEG, as well as the methods used to determine the gene essentiality. It is shown that the increase in prokaryotic records and eukaryotic records are mainly due to the widespread use of Tn-seq and CRISPR/Cas9, respectively (Figure 1).
2. We have developed built-in analysis modules by which users can perform various analyses, such as essential-gene distributions between bacterial leading and lagging strands, sub-cellular localization distribution, gene ontology and KEGG pathway enrichment analysis, and generation of Venn diagrams to compare and contrast gene sets between experiments.

## DETERMINATION OF ESSENTIAL GENES IN HUMANS

Genome-wide essentiality screenings have elucidated the molecular underpinnings of many biological processes in prokaryotes. However, limited knowledge has been gained regarding essential genes in human cells. Large-scale gene essentiality screenings across human cell types can reveal genes that encode factors for regulating tissue-specific cellular processes, and such screenings in cancer cells can disclose factors that determine cancer phenotypes, thus revealing important targets for cancer therapies. However, genome-wide inactivation of genes in human cells and the analysis of lethal phenotypes have been hampered by technical barriers.

One of the major breakthroughs in biotechnology has been the invention of CRISPR/Cas9 (CRISPR-associated RNA-guided endonuclease Cas9), which is a simple yet powerful tool for editing genomes (6,7). Cas9, an endonuclease, can be guided to specific locations within complex genomes by a guide RNA (gRNA). Cas9-mediated gene editing is simple and scalable, enabling the examination of gene functions at the systems level. Because of the ease and efficient targeting, CRISPR/Cas9 is described as being analogous to the ‘search’ function in a modern word processor (10). The invention of CRISPR/Cas9 has revolutionized the biological research in many fields, with essentiality screenings in human cells being no exception.

In 2015, three papers were published simultaneously reporting the genome-wide identification of essential genes among diverse human cell types (11–13). Wang *et al.* used the CRISPR-based approaches in analyzing multiple cell lines, and found tumor-specific dependencies on particular genes. The core-essential genes among these cell lines are enriched for genes with evolutionarily conserved pathways, with high expression levels, and with few detrimental polymorphisms in the human population (11). Analysis by Blomen *et al.* revealed a synthetic lethality map in human cells (12). Hart *et al.* used CRISPR-based approaches to screen for fitness genes among five cell lines, and consequently discovered 1580 human core fitness genes, and context-dependent fitness genes, that is, genes conferring pathway-specific genetic vulnerabilities in cancer cells (13).

The technology of CRISPR can be used on various cell types. Mair *et al.* used the CRISPR system to catalogue essential genes that are indispensable for human pluripotent stem cell fitness (14). Lu *et al.* determined genes essential for podocyte cytoskeletons based on single-cell RNA sequencing (15). Wang *et al.* used CRISPR in identifying essential oncogenes for hepatocellular carcinoma tumor growth (16). Arroyo *et al.* used a CRISPR-based screen and consequently identified essential genes for oxidative phosphorylation (17).

There is a major difference between cell-specific and organism-specific gene essentiality. That is, essential gene sets for human cells can be significantly different from those for human development. CRISPR technology, despite being powerful, cannot be used as a reverse genetics approach in humans for gene essentiality studies. Nevertheless, exome sequencing, another recent breakthrough, enables the identification of human essential genes *in vivo* (18).

Exome sequencing is considerably less expensive than whole-genome sequencing, and most Mendelian diseases are caused by genetic variations in protein-coding regions (exomes). The Exome Aggregation Consortium (ExAC) reported the exome sequences of 60 706 individuals, and the genetic diversity represents an average of one variant of every 8 bases of the exomes. Thus these variations are analogous to a genome-wide mutagenesis screening conducted in nature, similar to a transposon mutagenesis screening performed in the lab. Strikingly, 3230 genes contain near-complete depletion of protein-truncating variants, representing candidate human organism-level essential genes (18). Therefore, the number of essential gene in humans in DEG 15 has increased by >10-fold, primarily due to the use of CRISPR and exome sequencing technology (Table 1).

**Table 1.** Contents of DEG 15

Domain of life	Organism	No. of essential genomic elements		Method	Saturated	Reference	Note^a^
		Coding	Noncoding				
	*Acinetobacter baumannii* ATCC 17978	453	59	INSeq	Yes	(24)	
	*Acinetobacter baumannii* ATCC 17978	157	1	INSeq	Yes	(24)	In the mouse lung
	*Acinetobacter baylyi*	499		Single-gene knockout	Yes	(57)	Minimal medium
	*Aggregatibacter actinomycetemcomitans*	59		Tn-seq^b^	Yes	(58)	For coinfection with sympatric and allopatric microbes
	*Agrobacterium fabrum* str. C58	361	11	Tn-seq	Yes	(25)	
	*Bacillus subtilis*	261	2	Single-gene knockout	Yes	(59)	
Bacteria	*Bacillus thuringiensis* BMB171	516		Tn-seq	Yes	(60)	
	*Bacteroides fragilis*	550		Tn-seq	Yes	(61)	
	*Bacteroides thetaiotaomicron*	325		INSeq	Yes	(21)	
	*Bifidobacterium breve*	453		TraDIS	Yes	(62)	
	*Brevundimonas subvibrioides*	448		Tn-seq	Yes	(25)	
	*Brevundimonas subvibrioides* ATCC 15264	412	35	Tn-seq	Yes	(25)	
	*Burkholderia cenocepacia* J2315	383		TraDIS	Yes	(63)	
	*Burkholderia cenocepacia* K56–2	508		Tn-seq	Yes	(64)	
	*Burkholderia pseudomallei* K96243	505		TraDIS	Yes	(65)	
	*Burkholderia thailandensis*	406		Tn-seq	Yes	(66)	
	*Campylobacter jejuni*	233		Tn-seq	Yes	(67)	
	*Campylobacter jejuni* subsp*. jejuni* 81–176	384		Tn-seq	Yes	(68)	
	*Campylobacter jejuni* subsp*. jejuni* NCTC 11168	166		Tn-seq	Yes	(68)	
	*Caulobacter crescentus*	480	532	Tn-seq	Yes	(69)	
	*Escherichia coli*	620		Genetic footprinting	Yes	(70)	
	*Escherichia coli*	303		Single-gene knockout	Yes	(71)	
	*Escherichia coli*	379		CRISPR	Yes	(72)	
	*Escherichia coli* O157:H7	1265	37	Tn-seq	Yes	(26)	
	*Escherichia coli* ST131 strain EC958	315		TraDIS	Yes	(73)	
	*Francisella novicida*	396		Tn-seq	Yes	(74)	
	*Francisella tularensis* Schu S4	453		TraDIS	Yes	(75)	
	*Haemophilus influenzae*	667		Genetic footprinting	Yes	(76)	
	*Helicobacter pylori*	344		MATT	Yes	(77)	
	*Mycobacterium avium* subsp*. hominissuis* strain MAC109	230		Tn-seq	Yes	(78)	
	*Mycobacterium tuberculosis*	614		TraSH	Yes	(79)	
	*Mycobacterium tuberculosis*	774		Tn-seq	Yes	(80)	
	*Mycobacterium tuberculosis*	742	35	Tn-seq	Yes	(27)	
	*Mycobacterium tuberculosis*	461		Tn-seq	Yes	(81)	
	*Mycobacterium tuberculosis*	601		Tn-seq	Yes	(82)	
	*Mycoplasma genitalium*	382		Tn-seq	Yes	(19,83)	
	*Mycoplasma pneumoniae*	342	34	Tn-seq	Yes	(28)	
	*Mycoplasma pulmonis*	321		Tn-seq	Yes	(84)	
	*Neisseria gonorrhoeae* MS11	751		Tn-seq	Yes	(85)	
	*Porphyromonas gingivalis*	463		Tn-seq	Yes	(86)	
	*Porphyromonas gingivalis* ATCC 33277	281		Tn-seq	Yes	(87)	
	*Providencia stuartii* strain BE2467	496	25	Tn-seq	Yes	(88)	
	*Pseudomonas aeruginosa*	335		TraSH	Yes	(89)	
	*Pseudomonas aeruginosa*	117		Tn-seq	Yes	(23)	
	*Pseudomonas aeruginosa*	321		Tn-seq	Yes	(90)	
	*Pseudomonas aeruginosa* PAO1	336		Tn-seq	Yes	(91)	
	*Pseudomonas aeruginosa* PAO1	551		Tn-seq	Yes	(92)	
	*Ralstonia solanacearum* GMI1000	465		Tn-seq	Yes	(93)	
	*Rhodobacter sphaeroides*	493		Tn-seq	Yes	(94)	
	*Rhodopseudomonas palustris* CGA009	522		Tn-seq	Yes	(95)	
	*Salmonella enterica Typhimurium*	306	15	TraDIS	Yes	(29)	
	*Salmonella entericaserovar* Typhi	356		TraDIS	Yes	(20)	
	*Salmonella entericaserovar* Typhi Ty2	358	24	TraDIS	Yes	(29)	
	*Salmonella entericaserovar* Typhimurium	105		Tn-seq	Yes	(96)	
	*Salmonella entericaserovar Typhimurium* SL1344	353	23	TraDIS	Yes	(29)	
	*Salmonella* typhimurium	490		Insertion-duplication	Yes	(97)	
	*Shewanella oneidensis*	403		Transposon mutagenesis	Yes	(98)	
	*Sphingomonas wittichii*	579	32	Tn-seq	Yes	(30)	
	*Staphylococcus aureus*	302		Antisense RNA	No	(99,100)	
	*Staphylococcus aureus*	351		TMDH	Yes	(101)	
	*Staphylococcus aureus* subsp*. aureus* MRSA252	295		Tn-seq	Yes	(102)	
	*Staphylococcus aureus* subsp*. aureus* MSSA476	305		Tn-seq	Yes	(102)	
	*Staphylococcus aureus* subsp*. aureus* MW2	256		Tn-seq	Yes	(102)	
	*Staphylococcus aureus* subsp*. aureus* NCTC 8325	288		Tn-seq	Yes	(102)	
	*Staphylococcus aureus* subsp*. aureus* USA300 TCH1516	295		Tn-seq	Yes	(102)	
	*Streptococcus agalactiae* A909	317		Tn-seq	Yes	(103)	
	*Streptococcus mutans* UA159	197	6	Tn-seq	Yes	(104)	
	*Streptococcus pneumoniae*	113		Insertion-duplication	No	(105)	
	*Streptococcus pneumoniae*	133		allelic replacement mutagenesis	No	(106)	
	*Streptococcus pneumoniae*		72	Tn-seq	Yes	(31)	
	*Streptococcus pyogenes* MGAS5448	227		Tn-seq	Yes	(107)	
	*Streptococcus pyogenes* NZ131	241		Tn-seq	Yes	(107)	
	*Streptococcus sanguinis*	218		Single-gene knockout	Yes	(108)	
	*Streptococcus suis*	361		Tn-seq	Yes	(109)	
	*Synechococcus elongatus* PCC 7942	682	34	Tn-seq	Yes	(110)	
	*Vibrio cholerae*	789		Tn-seq	Yes	(111)	
	*Vibrio cholerae* C6706	343		Tn-seq	Yes	(112)	
	*Vibrio vulnificus*	316		Tn-seq	Yes	(113)	
Archaea	*Methanococcus maripaludis*	519		Tn-seq	Yes	(32)	
	*Sulfolobus islandicus* M.16.4	441		Tn-seq	Yes	(33)	
Eukaryotes	*Arabidopsis thaliana*	358		Single-gene knockout	No	(54)	
	*Aspergillus fumigatus*	35		Conditional promoter replacement	No	(114)	
	*Bombyx mori*	1006		CRISPR	Yes	(115)	
	*Caenorhabditis elegans*	44		Genetic mapping	No	(116)	
	*Caenorhabditis elegans*	294		RNA interference	No	(56)	
	*Danio rerio*	315		Insertional mutagenesis	No	(117)	
	*Drosophila melanogaster*	376		P-element insertion	No	(118)	
	*Homo sapiens*	2452		OMIM annotation^c^	No	(119)	
	*Homo sapiens*	1562		CRISPR	Yes	(14)	Stem cells
	*Homo sapiens*	1593		CRISPR	Yes	(14)	HAP1 cells
	*Homo sapiens*	1690		CRISPR	Yes	(120)	Core essential genes among 17 cell lines
	*Homo sapiens*	3230		Exome sequencing	Yes	(18)	
	*Homo sapiens*	2054		CRISPR	Yes	(12)	KBM7 cells
	*Homo sapiens*	2181		CRISPR	Yes	(12)	HAP1 cells
	*Homo sapiens*	1878		CRISPR	Yes	(11)	KBM7 cells
	*Homo sapiens*	1660		CRISPR	Yes	(11)	K562 cells
	*Homo sapiens*	1630		CRISPR	Yes	(11)	Jiyoye cells
	*Homo sapiens*	1461		CRISPR	Yes	(11)	Raji cells
	*Homo sapiens*	1196		CRISPR	Yes	(13)	A375 cells
	*Homo sapiens*	1892		CRISPR	Yes	(13)	DLD1 cells
	*Homo sapiens*	2196		CRISPR	Yes	(13)	GBM cells
	*Homo sapiens*	2073		CRISPR	Yes	(13)	HCT116 cells
	*Homo sapiens*	386		shRNA	Yes	(13)	HCT116 cells
	*Homo sapiens*	1696		CRISPR	Yes	(13)	HeLa cells
	*Homo sapiens*	2038		CRISPR	Yes	(13)	RPE1 cells
	*Homo sapiens*	92		Functional genomics	No	(15)	Podocytes
	*Homo sapiens*	79		CRISPR	Yes	(16)	Hepatocellular carcinoma
	*Homo sapiens*	191		CRISPR	Yes	(17)	K562 cells
	*Komagataella phaffii* GS115	753		Tn-seq	Yes	(121)	
	*Mus musculus*	435		Single-gene knockout	No	(53)	Embryonic lethality
	*Mus musculus*	1933		Single-gene knockout	No	(52)	Preweaning lethality
	*Mus musculus*	2136		MGI annotation^d^	No	(122)	
	*Plasmodium falciparum*	2680		transposon mutagenesis	Yes	(34)	
	*Saccharomyces cerevisiae*	1110		Single-gene knockout	Yes	(123)	Six conditions including minimal medium
	*Schizosaccharomyces pombe*	1260		Single-gene knockout	Yes	(124)	Rich medium

^a^Bacteria were cultured in rich media, unless otherwise indicated.

^b^Tn-seq is a method that performs saturated transposon mutagenesis followed by parallel sequencing to determine the transposon integration sites. Tn-seq has many variants under different names, such as insertion sequencing (INSeq), Transposon Directed Insertion Sequencing (TraDIS), high-throughput insertion tracking by deep sequencing (HITS), transposon sequencing, Microarray tracking of transposon mutants (MATT), Transposon site hybridization (TraSH), transposon mutagenesis followed by Sanger sequencing, transposon mutagenesis followed by genetic footprinting, transposon-site hybridization, Transposon-Mediated Differential Hybridisation (TMDH).

^c^OMIM: Online Mendelian Inheritance in Man (125).

^d^MGI: Mouse Genome Informatics (126).

## THE WIDESPREAD USE OF Tn-seq

Tn-seq technology has been successfully used in identifying essential genes in a large number of bacteria, and it has also been used in archaea and even a eukaryote. In comparison to the single gene knockout method, Tn-seq is less time-consuming and labor-intensive, because of the parallel nature in mutagenesis and insertion site determination. The invention of the Tn-seq method can date back to a study in which Venter and coworkers performed Sanger sequencing to determine transposon insertion sites (19) in 1999. In 2009, two technologies, high-density transposon-mediated mutagenesis and high-throughput sequencing, were mature, creating conditions that enabled Tn-seq to be invented (9). Many variants of Tn-seq were proposed, such as TraDIS (20), INSeq (21), HITS (22) and Tn-seq Circle (23). Here, we refer to these methods collectively as Tn-seq since they all involve transposon mutagenesis and sequencing.

Tn-seq has been widely used in identifying essential genes in bacteria. Figure 1A shows that since 2009, when DEG 5 was published (4), most bacterial essential genes have been determined by Tn-seq, and the proportion of essential genes that are determined by Tn-seq has been increasing ever since. This is not surprising given the powerfulness, ease of use, and the efficiency of Tn-seq in performing essentiality screening. Another advantage of Tn-seq is that it identifies not only essential protein-coding genes, but also non-coding genomic elements. For instance, by using Tn-seq, a large number of non-coding genomic elements have been determined in *Acinetobacter baumannii* (24), *Brevundimonas subvibrioides* (25), *Escherichia coli* O157:H7 (26), *Mycobacterium tuberculosis* (27), *Mycoplasma pneumonia* (28), *Salmonella entericaserovar* Typhimurium (29), *Sphingomonas wittichii* (30) and *Streptococcus pneumonia* (31).

In addition, Tn-seq has been used to determine essential genes in species other than bacteria. The methanogenic archaeon *Methanococcus maripaludis* S2 is an obligate anaerobic prokaryote that lives in oxygen-free environments. Sarmiento *et al.* used the Tn-seq method and identified 526 essential genes required for growth in rich medium, representing the first genome-wide gene essentiality screening in archaea (32). The second essentiality screening in archaea was conducted in *Sulfolobus islandicus*, and some archaea specific essential genes were identified (33). Moreover, Tn-seq was also used in identifying essential genes in a eukaryote. Severe malaria is caused by the apicomplexan parasite *Plasmodium falciparum*, a unicellular protozoan parasite of humans, and 680 genes were identified as essential for optimal growth of this parasite (34). Because of the widespread use of Tn-seq, the number of prokaryotic essential genes in DEG 15 has more than doubled compared to that of DEG 10 (Figure 1A).

## ANALYSIS MODULES

**Figure 2.**
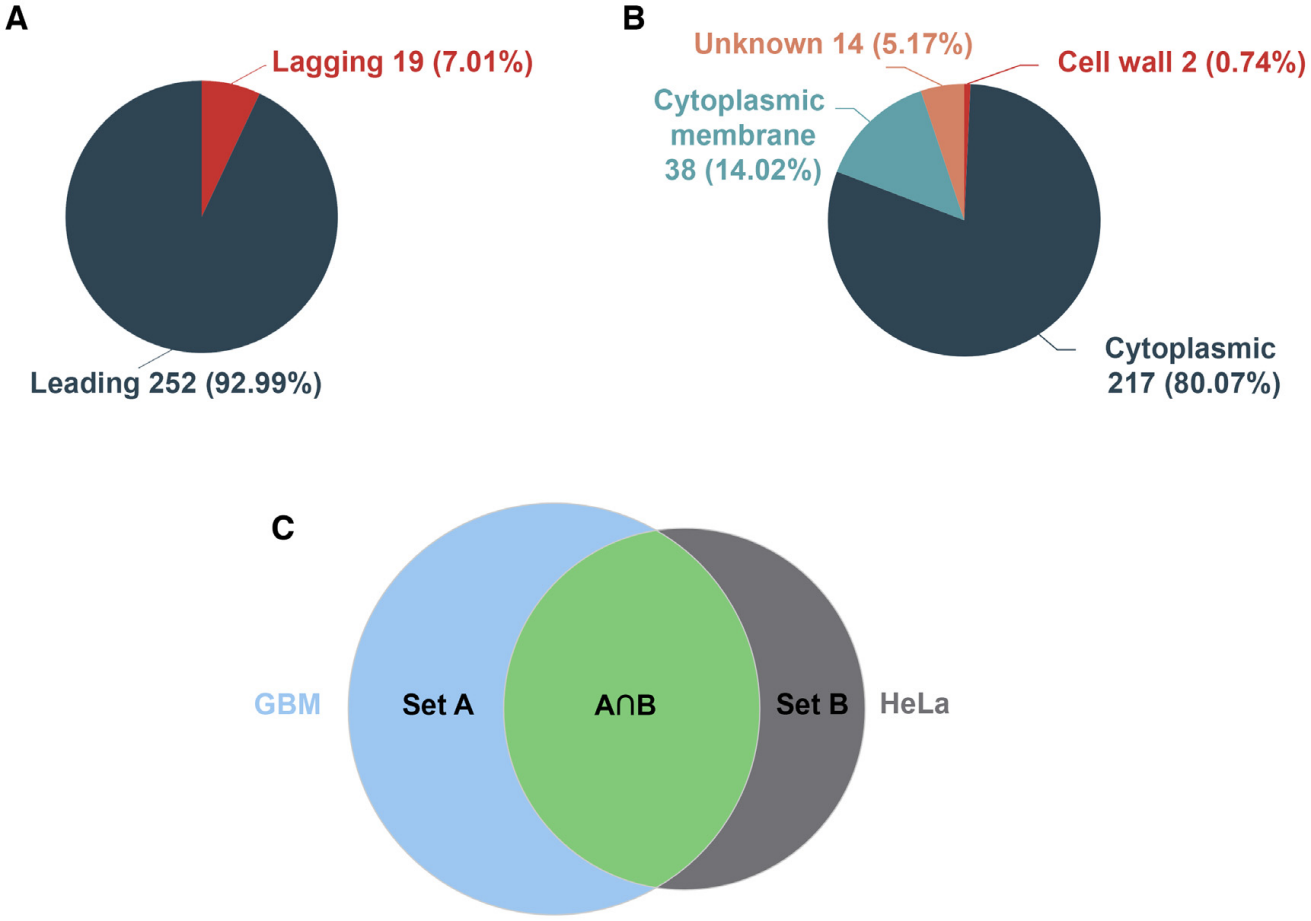
Screenshots of some analysis modules in DEG 15. (**A**) Distribution of essential genes between leading and lagging strands and (**B**) distribution of sub-cellular localizations of essential genes in the *Bacillus subtilis* genome. (**C**) A Venn diagram showing the intersection and the union between two datasets (GBM and HeLa cells). The diagrams are clickable to show a list of genes with detailed information.

To facilitate the use of DEG, we developed a set of analysis modules in the current release. Essential genes are preferentially situated in the leading strand, rather than the lagging strand (35), mainly because of the decreased mutagenesis pressure resulting from the head-on collisions of transcription and replication machineries in the leading strand (36). We obtained replication origins and determined leading *vs*. lagging strands using the DoriC database (37,38). Users can examine essential gene distributions between leading and lagging strands, and clicking the pie graph will display a list of genes in leading or lagging strands (Figure 2A).

Sub-cellular localization and operon information were obtained from the PSORTb v3.0 tool and the DOOR database, respectively (39,40). Clicking a species name, e.g. *Bacillus subtilis*, will display sub-cellular localization distributions of essential genes, and detailed gene information can be further examined by clicking on a particular cell compartment (Figure 2B). Other information includes orthologous groups, EC number (41), KEGG pathway (42) and GO (43), as determined by eggNOG-mapper (44). Users can analyze the GO distributions, and enriched GO terms powered by GOATOOLs (45), and enriched KEGG pathways, obtained using clusterProfiler package in R language (46). The analysis results, including strand bias distribution, sub-cellular distribution, and enrichment analysis of GO and KEGG pathways, are visualized with ECharts (47).

To analyze human essential genes, we developed a tool by which users can compare and contrast the essential gene sets between experiments, generate Venn diagrams to visualize the comparison, and obtain unions and intersections for the two gene sets by clicking the corresponding graph (Figure 2C). Furthermore, DEG 15 continues to provide customizable BLAST tools that allow users to perform species- and experiment-specific searches for a single gene, a list of genes, annotated or un-annotated genomes.

## FUTURE PERSPECTIVE

The identification of essential genes in both prokaryotes and eukaryotes has attracted significant attention over the past decade, largely because of the practical implications of these studies (2). Bacterial essential genes are attractive drug targets, as inhibiting these genes can suppress bacterial survival (48). Interest on essentiality screenings has also been boosted by synthetic biology, which aims to make an artificial self-sustainable living cell (49). The minimal gene set of a bacterium is considered a chassis for further addition of other parts with desirable traits. An increasing number of essentiality screens are being performed in a context-specific manner. For instance, essential genes for cancer cells can reveal cancer-specific cellular processes, which are targets for cancer drugs (50). Determination of essential genes of *A. baumannii* revealed genes required for its infection and survival in the lung (24). Moreover, another important direction is the prediction of gene essentiality using bioinformatic approaches, *e.g*., based on metabolic models (51). Therefore, because of the theoretical implications of the minimal-gene-set concept and its practical uses, it is expected that the essential gene identifications will continue to be further advanced.

Reverse genetics will continue to be indispensable for pinpointing gene functions. It is expected that single-gene knockout projects for the model organisms, such as mice (52,53) and *Arabidopsis thaliana* (54), will soon be completed. Multiple ways to manipulate gene expression are available, such as those based on TetR/Pip-OFF repressible promoter system (55) and RNA interference (56). From the aspect of technology, this is a golden era for essential-gene research, because of the availability of Tn-seq and CRISPR/Cas9. The two technologies enable the gene essentiality screenings in a wide range of cell types and species under diverse conditions. Therefore, we anticipate that the increase in the number of essential genes for many cell types under various conditions will be accelerated in the future. Therefore, we will continue to update DEG with high-quality human-curated data in a timely manner to keep pace with this rapidly developing field.

## DATA AVAILABILITY

DEG is accessible from essentialgene.org or tubic.org/deg. All DEG data is freely available to download.
